# Simultaneous Salt Rejection and Heat Localization Via Engineering Macrochannels in Morning Glory‐Shaped 3D Evaporator

**DOI:** 10.1002/advs.202405639

**Published:** 2024-08-29

**Authors:** Zhengyi Mao, Yicheng Han, Junda Shen, Lei Zhang, Youneng Xie, Jiahua Liu, Haikun Wu, Zhen Yu, Xiaoguang Duan, Yaoxin Zhang, Jian Lu

**Affiliations:** ^1^ CityU‐Shenzhen Futian Research Institute Shenzhen 518045 China; ^2^ Department of Mechanical Engineering City University of Hong Kong 83 Tat Chee Avenue Kowloon Hong Kong 0000 China; ^3^ Department of Material Science and Engineering City University of Hong Kong 83 Tat Chee Avenue Kowloon Hong Kong 0000 China; ^4^ School of Chemical Engineering and Advanced Materials University of Adelaide Adelaide 5005 Australia; ^5^ China‐UK Low Carbon College Shanghai Jiao Tong University Shanghai 200025 China; ^6^ Hong Kong Branch of National Precious Metals Material Engineering Research Centre City University of Hong Kong 83 Tat Chee Avenue Kowloon Hong Kong 0000 China

**Keywords:** convection, energy management, salt harvesting, salt rejection, solar desalination

## Abstract

Solar desalination is a promising solution for alleviating water scarcity due to its low‐cost, environmentally friendly, and off‐grid capabilities. However, simultaneous salt rejection and heat localization remain challenging, as the rapid salt convection often results in considerable heat loss. Herein, this challenge is overcome via a facile design: i) isolating high‐temperature and high‐salt zones by rationally designing morning glory‐shaped wick structures and ii) bridging high‐salt zones and bulk water with low‐tortuosity macrochannels across low‐temperature surfaces. The salinity gradient in the macrochannels passively triggers convective flow, facilitating the rapid transfer of salt ions from the high‐salt zone to the bulk water. Meanwhile, the macrochannels are spatially isolated from the high‐temperature zone, preventing heat loss during salt convection and thereby achieving a high evaporation rate (≈3 kg m^−2^ h^−1^) and superior salt rejection even in highly concentrated real seawater. This work provides new insights into salt rejection strategies and advances practical applications for sustainable seawater desalination.

## Graphical abstract

By rationally engineering a biomimetic morning glory‐shaped evaporator with salt‐heat isolation, and incorporating the macrochannels to enable fast salt convection through the cool outer surface, the design simultaneously achieves a high evaporation rate and superior salt rejection. This design is also demonstrated to be effective when tested with real concentrated seawater.

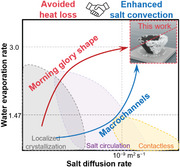

## 1 Introduction

Clean water has always been essential to human well‐being.^[^ ^1^ ^]^ Population growth, urbanization, industrialization, and climate change have undoubtedly intensified water scarcity, leaving millions of people without access to safe drinking water.^[^ ^2^, ^3^, ^4^ ^]^ Interfacial solar water evaporation is considered one of the most sustainable desalination technologies for relieving fresh water scarcity due to its unique advantages of low cost, off‐grid capability, and zero carbon footprint.^[^ ^5^, ^6^, ^7^, ^8^, ^9^, ^10^, ^11^, ^12^ ^]^ In recent years, numerous materials,^[^ ^13^, ^14^, ^15^, ^16^, ^17^, ^18^, ^19^ ^]^ and structures^[^ ^20^, ^21^, ^22^, ^23^, ^24^, ^25^, ^26^ ^]^ have been developed for achieving high‐efficiency water evaporation. Among these materials and structures, 3D evaporators are emerging as promising designs, as they can capture additional energy from the surroundings and contribute greatly to evaporation, yielding an evaporation rate that exceeds the theoretical maximum value of 1.45 kg m^−2^ h^−1^.^[^ ^27^, ^28^, ^29^, ^30^, ^31^ ^]^ However, beyond the evaporation rate, the challenge of salt fouling has gained increasing attention. Due to the slow diffusivity of salt in water (≈10^−9^ m^2^ s^−1^), there is significant salt accumulation during continuous operation. The accumulated salt seriously compromises sunlight absorption and blocks water transport channels, deteriorating evaporation performance and ultimately leading to system failure.^[^ ^32^ ^]^

**Figure 1:**
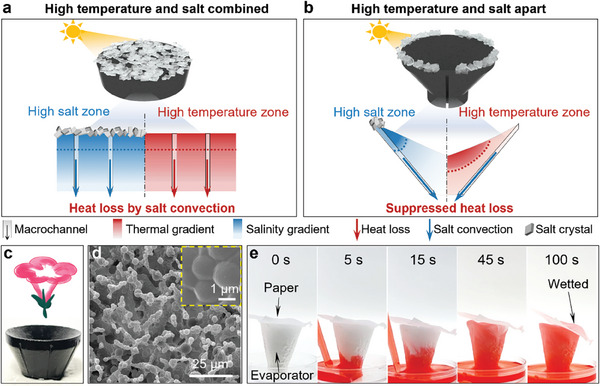
Design and characterization of the MG evaporator. a) Schematic of heat localization and salt rejection in a conventional convection‐based evaporator. The high‐temperature and high‐salt concentration zones are consistently overlapping, resulting in significant heat loss during salt convection through the macrochannels. b) Schematic of heat localization and salt rejection in our evaporator. The high‐temperature and high‐salt zones are segregated, where the interior surface is hot, and the outer surface and edges are cold. Salt crystallizes locally at the edges, while the macrochannels on the cold side efficiently transfer salt from the high‐salt zone with minimal heat loss. c) Images of morning glory flower and our evaporator. d) SEM images of the evaporator. The inset shows the PU skeleton. e) Water transport in the MG evaporator (height: 2 cm).

Recently, innovative strategies have been proposed for achieving long‐term solar desalination, which can be classified into three general categories. i) Separation of solar absorbers and salt water (e.g., contactless evaporation and Janus structures).^[^ ^33^, ^34^, ^35^, ^36^, ^37^ ^]^ This design prevents brine from reaching the light absorption layer, thus avoiding surface salt accumulation. However, the evaporation performance is hindered by the rapid heat loss from the photothermal layer to the environment or the bulk water, decreasing the energy conversion efficiency. ii) Localized salt crystallization.^[^ ^38^, ^39^, ^40^, ^41^, ^42^ ^]^ Zhang's group^[^ ^43^ ^]^ reported a 2D evaporator with edge‐preferential crystallization, which they obtained by adjusting the brine transportation pathway. Despite the excellent salt rejection capability, the evaporation rate of the evaporator is relatively low (≈1.4 kg m^−2^ h^−1^). iii) Convective flow‐intensified salt transfer. Engineering high‐throughput macrochannels in the wick structure to enhance fluid convection is believed to be the simplest method for achieving salt rejection.^[^ ^44^, ^45^, ^46^, ^47^, ^48^, ^49^ ^]^ By inducing low‐tortuosity macrochannels in the wick structure to bridge the high‐salt zone and bulk water, it is possible to strengthen the water supply and accelerate salt ion transportation (**Figure** **1**a). However, the fluid exchange between the high‐salt zone and the bulk water not only removes salt, but also causes significant heat loss and leads to a low evaporation rate due to the coincidence of the high‐temperature and high‐salt zones. Thus, simultaneous salt rejection and heat localization remain a grand challenge for interfacial solar water evaporation.

Herein, we present a morning glory‐shaped (MG) 3D evaporator with macrochannels on the outer surface that can simultaneously achieve superior salt resistance and a high evaporation rate. The spatial segregation of high‐salt and high‐temperature zones was achieved by engineered design of the 3D structure of the MG evaporator. The edge region exhibits a large air‐evaporator interface, facilitating the escape of vapor and the localized cooling. The lower positioning of the middle region increases the distance for vapor escape and diminishes the convective promotion of vapor escape. This leads to elevated temperatures in the middle of inner surface. Simultaneously, the 3D structural configuration of the MG evaporator promotes the preferential transportation of salt from the center to the edge, creating a high‐salt zone in the cooler edge region. This achieves the separation of the high‐salt zone from the high‐temperature zone. By coupling engineered capillary wick structures and macrochannels, buoyancy‐driven natural convection is triggered to promote salt transportation. As shown in Figure 1b, the high‐throughput macrochannels on the outer surface connect the high‐salt zone to the bulk water, initiating buoyant flow due to the salt gradient and thereby rapidly reflowing the salt to the bulk water. Moreover, isolating the high‐salt and high‐temperature zones suppresses the convective energy loss. With the inner surface having a high temperature and the outer shell and edges having low temperatures, the temperature difference between the buoyant flow and bulk water is reasonably lower than that of conventional convective evaporators. Hence, our design avoids significant energy losses during efficient convective salt rejection. As a result, the MG evaporator with macrochannels can stably operate during the desalination of highly concentrated brine while achieving a high evaporation rate of ≈3 kg m^−2^ h^−1^. Besides, our strategy demonstrates applicability when treating real, highly concentrated seawater (>10 wt.%), revealing its significant potential for practical brine desalination.

## 2 Results and Discussion

### 2.1 Design and Mechanism of the Biomimetic Evaporator

To prepare the MG evaporator, readily available freeze‐dried polyurethane (PU) and carbon black were selected as the porous matrix and photothermal materials, respectively. The self‐prepared PU sponge showed excellent water absorbing speed, mechanical durability, and low cost, which are for practical applications.^[^ ^50^ ^]^ The preparation process is shown in Figure S1 (Supporting Information). The mixed PU and carbon black aqueous solution was poured into a customized morning glory‐shaped silicon mold and frozen at −80 °C. After freeze‐drying to remove the ice crystals, a porous MG evaporator with macrochannels at the outer surface is obtained (Figure 1c). The evaporator exhibits a highly interconnected porous structure, which facilitates light trapping and water transportation (Figure 1d). A blended PU skeleton can be observed, providing the evaporator with mechanical durability. The prepared evaporator can rapidly absorb an 800 µL water droplet within 0.1 s due to its high affinity for water (Figure S2, Supporting Information). Moreover, the evaporator can pump water to a height of 20 mm in 45 s and fully wet the top paper, demonstrating the strong capillary force for water transfer (Figure 1e). The superior mechanical durability is proven by cyclic compression tests. After 100 compressions at 80% strain, the PU/carbon black sponge can be recovered to its original size without deterioration (Figure S3, Supporting Information). To measure the salt rejection and evaporation performance of our evaporator, the bottom part evaporator was inserted into an expanded polystyrene (EPS) foam for floating (Figure S4, Supporting Information).^[^ ^51^ ^]^

### 2.2 Heat Localization and Evaporation Performance

**Figure 2:**
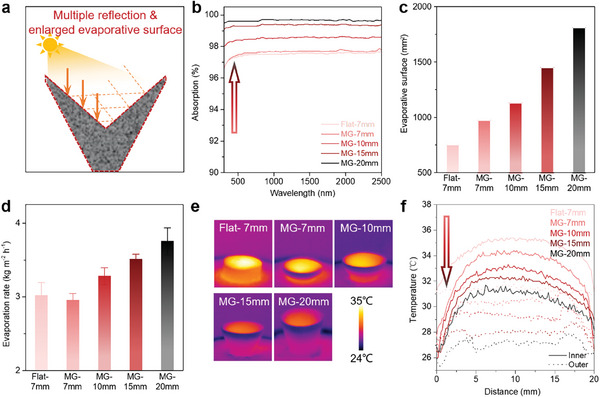
Heat localization and evaporation characteristics of MG evaporators. a) Enhanced light trapping and evaporative surface of the MG evaporator. b) Light absorption characteristics of the MG evaporators with different heights. c) Evaporative surface areas of different evaporators. d) Evaporation rates of the evaporators. e) IR images of different evaporators under one sun illumination. f) Outer and inner surface temperatures of the evaporators in e.

To demonstrate the effect of MG structure on the heat localization and evaporation performance, a flat evaporator with a height of 7 mm and MG evaporators with heights of 7, 10, 15, and 20 mm were prepared (Figure S5, Supporting Information). The MG structure shows better light trapping ability than the flat sample due to the absorption of light that is reflected multiple times (**Figure** **2**a). Simultaneously, the MG evaporators exhibit a larger air–evaporator interfaces for vapor generation while using fewer materials than the flat evaporators (Figure S6, Supporting Information), benefitting practical applications. The light absorption property was evaluated by ultraviolet (UV)‒visible (Vis)–near‐infrared (NIR) absorption spectra in the range of 250–2500 nm (Figure 2b). The MG evaporator with a height of 7 mm shows a light absorption level of 97.4%, which is slightly higher than that of the flat evaporator with the same height (97.2%). With further increases in the height of the MG evaporator, the light absorption significantly increases to 99.6%, which strongly demonstrates the superiority of the engineered 3D structure for light trapping. The light absorption performance of the MG evaporator is comparable to that of the recently reported structured light‐absorbing materials. (Figure S7, Supporting Information). Subsequently, we calculated the evaporative surfaces of different evaporators (Figure 2c). The evaporative surfaces of the flat and MG samples with heights of 7 mm are 753.6 mm^2^ to 972.9 mm^2^, respectively, representing an improvement by ≈30%. This enlarged surface area facilitates water molecules at the interface to gain energy and escape to the air, resulting in a high evaporation rate.^[^ ^52^ ^]^ The evaporation performance of the as‐prepared evaporators was measured under one sun illumination (Figure 2d). The evaporation rate increases from ≈2.9 kg m^−2^ h^−1^ for the 7 mm sample to 3.75 kg m^−2^ h^−1^ for the MG sample with a height of 20 mm, surpassing that of most previously reported salt‐rejection solar evaporators (i.e., localized salt crystallization based salt rejection^[^ ^39^, ^43^, ^53^ ^]^ and convective flow‐based salt circulation^[^ ^29^, ^44^, ^45^, ^54^, ^55^ ^]^). This enhancement occurs due to the engineered 3D architecture, which increases the light absorption level and evaporative surface area. Additionally, the water evaporation enthalpy is reduced by the materials, further contributing to this excellent evaporation rate (Figure S8, Supporting Information). Besides, the 3D structure of the MG evaporator enables efficient light capture, resulting in a high evaporation rate even under various illumination angles (Figure S9, Supporting Information). This observation highlights its practical potential when exposed to real sunlight.

Figure 2e illustrates the infrared (IR) temperature distribution of the prepared evaporators. The flat evaporator exhibits a high‐temperature zone at its top surface, which coincides with the high‐salt concentration zone, as depicted in Figure 1a. Conversely, the MG evaporator displays a distinct temperature profile with a concentrated hot zone at its inner surface, while the edge and outer surface exhibit reduced temperatures. This temperature distribution results in the separation of the high‐temperature and high‐salt zones within the MG evaporator. The temperature at the high‐salt zone of the flat evaporator is 35.4 °C, exceeding that of the MG evaporator with the same height by 7.7 °C. When the height of the MG evaporator increases to 20 mm, the temperatures at the edge and outer surface decrease to 24 and 26.4 °C, respectively (Figure 2f). The inner surface of the MG evaporator exhibits a gradient temperature distribution, which arises from the unique shape of the MG evaporator. The lower positioning of the middle region within the MG evaporator increases the distance for vapor escape. Simultaneously, the convective flow induced by evaporation at the boundary region fails to reach the central portion of the inner surface,^[^ ^56^ ^]^ leading to a reduced efficiency in vapor removal (Figure S10, Supporting Information). The accumulation of vapor results in elevated local humidity and subsequently higher temperatures. At the same time, the boundary region displays a larger interfacial surface area that facilitates efficient vapor escape, thereby promoting cooling through latent heat and radiation. This, in turn, leads to the gradient temperature distribution at the inner surface. The relatively low temperatures observed at the outer surface can be primarily attributed to the absence of direct illumination, while the evaporation‐induced cooling effect further enhances the temperature decline. Thus, by reducing the temperature difference between the high‐salt zone and the bulk water from 10.4 to 1.4 °C, the fluid convection‐induced heat loss through the macrochannel can be sharply diminished.

### 2.3 Localized Salt Precipitation and Convection

The salt resistance performance of the MG evaporators was measured at 20 wt.% NaCl solution. After 2 h of continuous desalination, the edge and outer surface of all evaporators were covered by salt crystals, which deteriorated the desalination performance (Figure S11, Supporting Information). To solve this problem, we designed macrochannels on the outer surface of the evaporators to facilitate the rapid transfer of salt from the evaporative surface to the bulk water. As a proof of concept, three types of evaporators were prepared, including a flat evaporator, a MG evaporator, and MG evaporators with macrochannels (height = 10 mm, Figure S12, Supporting Information).

**Figure 3:**
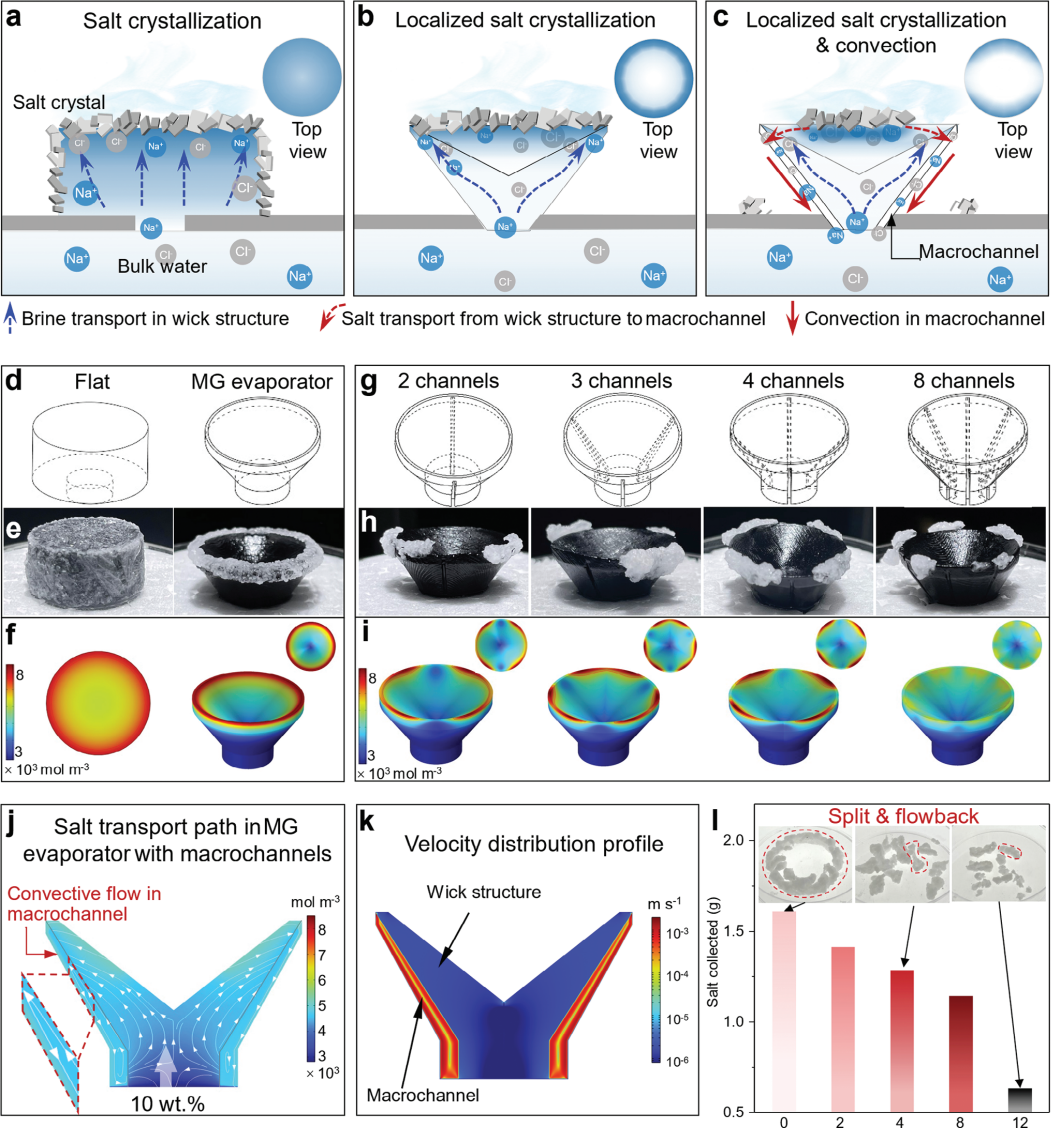
Localized salt crystallization and convection‐induced salt rejection. a) Illustration of salt crystallization on a flat sample. b) Localized salt crystallization on a MG evaporator without macrochannels. c) Localized salt precipitation and convection on a MG evaporator with macrochannels. d) Structure of flat and MG evaporators. e–f) Experimental and simulation results of salt crystallization on a flat sample and a MG evaporator. g) Structures of MG evaporators with 2, 3, 4, and 8 macrochannels. The macrochannels are circular arrays on the outer surface of the evaporator. h–i) Experimental and simulation results of salt crystallization on the evaporators in (g). j) Simulated salt transport pathway of the MG evaporator with two macrochannels. k) Velocity distribution profile of the MG evaporator with two macrochannels. l) Harvested salt crystals from MG evaporators with different numbers of macrochannels after 24 h of evaporation in 10 wt.% NaCl solution.

**Figure** **3**a–c shows the salt crystallization mechanisms of the prepared three evaporators. The evaporative surface of the flat evaporator was fully covered by salt, while salt only crystallized on the edge of MG evaporator. This is because of the different capillary penetration in the flat and MG evaporators. The flat evaporator passively absorbs brine from the bulk water and transports it to the evaporative surface. Continuous evaporation increases the salt concentration, resulting in salt crystallization and accumulation on the surface (Figure 3a). In comparison, the structural design of the MG evaporator enables preferential salt transport from the center to the edge (Figure 3b). Figure S13 (Supporting Information) presents the simulated salt transport paths of the MG evaporator and flat evaporator, respectively. Despite the salt ions convectively moving with the passive fluidic flow in both evaporators, the engineered shape of the MG evaporator leads to an edge‐preferential capillary flow, which results in localized salt accumulation at the edge of the MG structure. In addition, the low temperature on the edge generates great tension,^[^ ^57^ ^]^ accelerating the transport of brine to the edge. According to classical nucleation theory, heterogeneous nucleation tends to occur at low temperatures, which promotes edge‐preferential salt crystallization.^[^ ^58^ ^]^ However, the salt crystals formed at the edges will bond to the evaporator and gradually cover the evaporating surface, significantly compromising the evaporation rate.

We then designed the macrochannel on the outer surface of the MG evaporator to connect the high‐salt zone with the bulk water (Figure 3c). The salinity gradient between the high‐salt zone and the bulk water can passively trigger natural convection in the macrochannels, resulting in the reflow and splitting of the precipitated salt crystals.^[^ ^44^ ^]^ In addition, the adequate water supply induced by the macrochannel weakens the binding force between the split salt crystals and the evaporator.^[^ ^43^ ^]^ Consequently, salt particles suspended at the edge spontaneously detach due to gravity, achieving the long‐term stable desalination performance and providing an opportunity for harvesting valuable mineral resources from the desalination process. Figure 3d–f shows the experimental and simulation results of the flat and MG evaporators after 4 h of evaporation in 10 wt.% NaCl solution under one sun. The salt precipitates on the whole evaporation surface of the flat sample, transforming the surface color from black to white. Whereas salt only precipitates on the edge of the MG evaporator, demonstrating successful localized salt crystallization. The experimental results are in good agreement with the simulation results (Figure 3f), suggesting that salt crystallization can be precisely predicted by the computational method. Noted that, the tortuosity of the macrochannel is crucial for the salt convection. The low tortuosity macrochannel enables the fast salt transfer from the edge to the bulk water (Figure S14, Supporting Information).

To prove the effects of the macrochannel on fluid splitting and convection, four MG evaporators with 2, 3, 4, and 8 macrochannels arranged in a circular array on their outer surfaces were prepared (Figure 3g). Figure 3h,i display the precipitated salt crystals obtained from the experimental and simulated evaporation processes, respectively. In comparison to the MG evaporator without a macrochannel in Figure 3e, the circular‐shaped salt crystal zone is split into several parts due to the presence of macrochannels. By increasing the number of macrochannels from 2 to 8, the simulated area reaching the salt saturation point on the surface decreases and is divided into corresponding numbers of pieces. Figure 3j and Figure S15 (Supporting Information) show the salt transport path under the influence of macrochannels, revealing that buoyancy‐driven convection rapidly reflows the salt from the zone with a high salt concentration to the bulk water. Notably, this buoyancy‐driven natural convection results from density variation as salinity increases (Figure S16, Supporting Information). The brine with high downstream salinity flows downward with gravity, bringing salt ions to the bulk water. To characterize the contribution of convection and diffusion on salt transportation, the Peclet number (as the ratio between the convective transport rate and diffusive transport rate) proportional to the flow velocity is employed. Compared to the velocity in the wick structure, convection flow‐dominated salt transportation is observed in the marcochannels (Pe≫1) with a much higher velocity (Figure 3k). This result demonstrates that convective flow within the macrochannels significantly contributes to the rapid reflow of salt. The accumulated salts reflow into the macrochannels in a convective manner, significantly improving the salt rejection capability. Moreover, the sufficient water supply by the channels weakens the binding forces of the salt crystal and the evaporator, which can cause the salt crystals to fall from the edge of the evaporator (Video S1, Supporting Information). The salt crystals collected after a continuous 24 h evaporation process provide further evidence of the split and rejection phenomenon caused by the macrochannels. Both the size and weight of the collected salt crystals decrease as the number of macrochannels increases (Figure 3l).

The effectiveness of minimal heat loss was confirmed by monitoring the temperatures of the bulk water beneath the MG evaporator and the MG evaporator with eight macrochannels (Figure S17, Supporting Information). After 4 h of continuous evaporation under one sun, the temperature of the bulk water in both groups is approximately the same as the environmental temperature, demonstrating the negligible heat loss by convection in macrochannels. The heat loss in macrochannels was quantitatively calculated. The convection flow in the eight macrochannels slightly increases the heat loss to the bulk water by 0.0025 W, which accounts for 0.8% of the total solar energy input (Figure S18, Supporting Information). The heat transfer simulation result also demonstrates that the reduced heat loss by our design (Figure S19, Supporting Information). Moreover, we compared the evaporation rates of MG evaporators with varying numbers of macrochannels (0, 4, 8, and 12). The increase in the number of macrochannels does not decrease the evaporation rate, providing further evidence of the minimal heat loss caused by the macrochannels (Figure S20, Supporting Information). However, the greater the number of macrochannels, the thinner the edge becomes, resulting in a noticeable increase in manufacturing difficulty. Compared with conventional convection salt‐resistant structures, the heat loss through the macrochannels is significantly reduced (Figure S21, Supporting Information).

### 2.4 Modeling of Salt Transport in the Macrochannel

A multiphysics fluid flow model was developed to understand the salt transportation process in 3D evaporators. To distinguish the wick zone and marcochannels, the laminar fluidic flow is consistently governed by mass conservation and the Brinkman equation while neglecting the inertial term:

(1) $$\frac{\partial }{\partial t}\left(\varepsilon \rho \right)+\nabla \cdot \;\left(\rho u\right)=\;0$$

(2) $$\begin{array}{ccc} \rho \;\frac{\partial u}{\partial t} & = & \;-\nabla p+\nabla \cdot \left\{\frac{\mu }{\varepsilon }\left[\nabla u+{\left(\nabla u\right)}^{T}-\frac{2}{3}\left(\nabla \cdot u\right)I\right]\right\}\\ & & -\frac{\mu }{\kappa }u+\rho g\left[1-\alpha \left(T-T_{\infty }\right)\right] \end{array}$$

where ρ, **u,** and *p* represent the density of brine, the velocity vector, and the pressure, respectively. Gravity is induced by the gravitational constant **g**. The porous medium of the wick is represented by the porosity ε and permeability κ. In macrochannels, the viscous loss induced by porous media is avoided by reducing the Brinkman equation to the Navier‒Stokes equation as ε and κ vanish. The temperature field and salt transportation are determined by energy conservation and convection–diffusion equations, respectively:

(3) $$\rho C_{p}\left(\frac{\partial T}{\partial t}+u\cdot \nabla T\right)+\nabla \cdot q\;=\;0$$

(4) $$\frac{\partial \varepsilon c}{\partial t}+u\cdot \nabla c-\nabla \cdot \varepsilon \tau D_{L}\nabla c\;=\;0$$

Notably, the brine density varies with respect to the salt concentration c and temperature T, initiating buoyancy‐driven flows. To capture these natural convection flows, Equations (3) and (4) are coupled with the fluid flow model (Equations (1) and (2)). In this study, energy induced by incident solar flux is applied on the top surface of the evaporator. This uniform heat flux accordingly results in the evaporation of the brine–air interface and the accumulation of salt. Moreover, the bottom surface contacting the reservoir provides a fresh water supply (brine at the salt concentration in seawater at room temperature).

### 2.5 Long‐Term Stability

**Figure 4:**
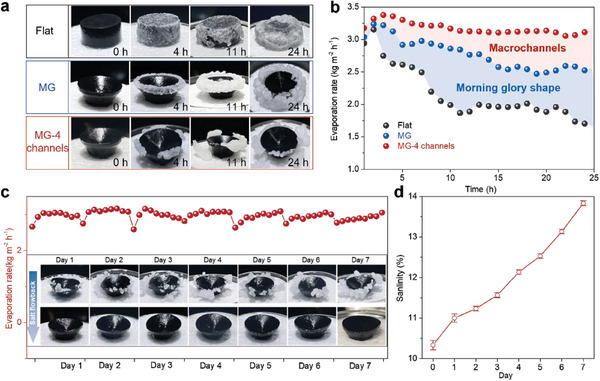
Long‐term stability of our evaporator in 10 wt.% NaCl solution. a–b) Optical images and evaporation rates of the flat evaporator, MG evaporator, and MG evaporator with four channels during 24 h of continuous operation. c) Evaporation rate during the 7‐d cyclic evaporation test. (Inset: optical images of the MG evaporator with 12 macrochannels during the cyclic test.) d) Salinity of the bulk water underneath the evaporator during the cycling experiment. All samples are prepared with heights of 10 mm.

To evaluate the effects of the engineered MG‐shaped wick structures and macrochannels on the salt rejection and evaporation performance, long‐term evaporation tests were conducted in 10 wt.% NaCl solution. After 24 h of continuous operation under one sun, the flat evaporator is thoroughly encrusted with salt crystals (**Figure** **4**a). This phenomenon significantly reduces the rate of evaporation from 3.15 kg m^−2^ h^−1^ to 1.7 kg m^−2^ h^−1^ (Figure 4b). For the MG evaporator, the salt precipitates at the edge of the evaporator, slightly reducing the evaporation rate from 3.23 kg m^−^ h^−1^ to 2.52 kg m^−2^ h^−1^. This finding demonstrates that edge‐preferential salt crystallization can isolate salt from the majority of the evaporation surface, thereby avoiding the cessation of evaporation. In comparison, the salt crystal is split into four parts, and it falls off automatically under gravity for the MG evaporator with four macrochannels. The evaporation rate remains steady at 3.1 kg m^−2^ h^−1^ during long‐term operation. These results illustrate that the MG structure facilitates localized salt crystallization, while the macrochannels split and transfer the salt to the bulk water without inducing heat loss. A 7‐d cycling experiment was conducted to further confirm the long‐term stability of the evaporator. In each cycle, the evaporator was operated for 9 h under one sun, simulating typical daily natural sunshine irradiation in Hong Kong. The 10 wt.% NaCl solution was periodically replenished to maintain a constant distance between the evaporator and light source. During the 7‐d cycling test, the evaporator rate stabilizes at approximately 3 kg m^−2^ h^−1^ without degradation (Figure 4c). This stability is attributed to the engineered 3D structure and macrochannels, which facilitate the fast transport of salt ions and prevent salt crystal blockage of the evaporative surface. The inset in Figure 4c shows the salt crystallization and flowback process. After operation under one sun, the salt precipitates locally at the edge of the evaporator, splitting into several small parts, while some salt crystals fall off on the floating foam due to gravity. After turning off the light, the precipitated salts can flow back to the bulk water, driven by the strong water supply of the highly interconnected porous structure and the macrochannel. The salinity of the NaCl solution increases from 10.3 wt.% to 13.9 wt.% due to the flowback process of the salt ions (Figure 4d).

### 2.6 Salt Rejection in Real Concentrated Seawater

**Figure 5:**
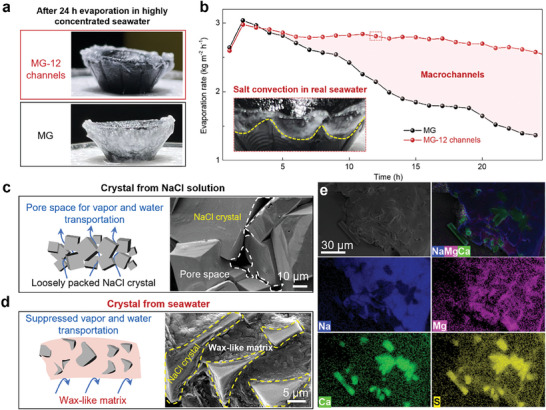
Salt rejection in real seawater. a) Optical images of the MG evaporator and the MG evaporator with 12 macrochannels during 24 h of operation in highly concentrated seawater (10.4 wt.%). b) Evaporation rates of the MG evaporator and the MG evaporator with 12 macrochannels. Inset shows the salt crystal on MG evaporator with 12 macrochannels after 12 h of evaporation. Schematic illustration and SEM images of accumulated salt crystals collected from c) pure NaCl solution and d) concentrated seawater. e) SEM images of the salt crystals from concentrated seawater and the corresponding ion distributions (NaCl and CaSO_4_ crystals, MgSO_4_).

The evaporation performance in real‐world concentrated seawater brine is of great significance for the field of solar desalination, but it has rarely been proposed thus far.^[^ ^29^, ^53^, ^59^ ^]^ Salt rejection in highly concentrated seawater is challenging due to the complex salt crystallization behavior that occurs in natural seawater, which contains a greater variety of constituent elements than pure NaCl solution.^[^ ^53^ ^]^ When tested in pure NaCl solution, the water evaporation remains stable for a long period. However, when operating in real concentrated seawater brine, the water evaporation level declines quickly. This difference can be attributed to the significant disparity in salt crust structures between pure NaCl solution and real seawater. To demonstrate the practicality of our design, we conducted a continuous operation to measure the evaporation performance of the MG evaporator and the MG evaporator with 12 macrochannels in highly concentrated seawater (10.4% salinity). Unlike the ring‐shaped salt crust formed from the evaporation of 10 wt.% pure NaCl solution, the MG evaporator in concentrated seawater exhibits a dense salt film, ultimately covering the entire unit (**Figure** **5**a). The resulting precipitation of salt crystals sharply decreases the evaporation rate by 1.67 kg m^−2^ h^−1^ (Figure 5b), which is over 2.3 times higher than that measured for pure NaCl solution in Figure 4b (0.71 kg m^−2^ h^−1^). In contrast, in the MG evaporator with macrochannels, salt only precipitates at the edge and exhibits a jagged shape (inset in Figure 5b). The macrochannel enables the salt ions to flow quickly into the underlying seawater, thereby reducing salt crystallization near the macrochannels. Consequently, the evaporation rate of the MG evaporator with 12 macrochannels remains stable during continuous operation. This finding demonstrates that convection‐induced salt rejection is applicable for real and concentrated seawater.

To illustrate the difference in evaporation performance when treating pure NaCl solution versus natural seawater, salt crystals formed from both solutions with the same concentration (≈10 wt.%) were investigated. Figure 5c shows the salt crust obtained from the evaporation of pure NaCl solution, where the cubic salt crystals exhibit clean surfaces, and the loosely packed salt permits water transport and vapor escape. However, the salt crust from the concentrated seawater shows a dense structure. In addition to the cubic NaCl crystals, some clubbed crystals can be observed, and the gap space between the crystals is filled with a wax‐like matrix (Figure 5d). Energy‐dispersive X‐ray spectroscopy (EDS) analysis reveals that the clubbed crystals are calcium sulfate, sparsely located between the NaCl crystals (Figure 5e). Magnesium can be identified in the wax‐like substances, filling the entire pore space around the NaCl and CaSO_4_ crystals and forming a dense salt crust. Thus, the operation of the MG evaporator in concentrated seawater is halted by the formation of the dense salt crust. However, for the MG evaporator with macrochannels, the strong convection in the macrochannels allows for the backflow not only NaCl but also calcium and magnesium ions, preventing the precipitation of dense salt crust on the main evaporation surface.

To further prove the salt rejection capability of our evaporator in real seawater, we continuously evaporated 110 mL of seawater. The evaporator was fabricated with one section containing six macrochannels and another section without macrochannels, as shown in Figure S22 (Supporting Information). After 150 h of continuous operation under one sun, the underlying seawater is nearly completely evaporated. The evaporator displays an intriguingly asymmetric distribution of the salt crystals, where the section containing macrochannels exhibits localized and fragmented salt crystals. Conversely, the section without macrochannels is entirely covered by the salt crystal (Figure S23, Supporting Information).

### 2.7 Outdoor Test

**Figure 6:**
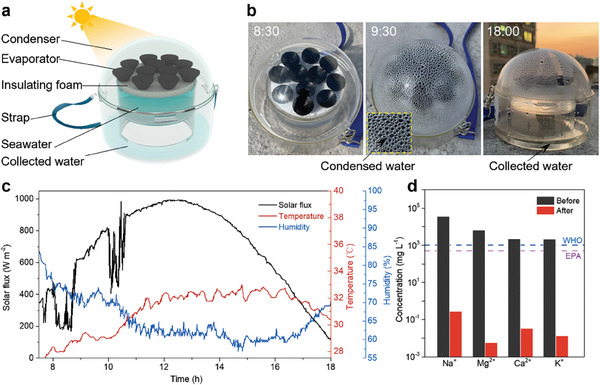
Outdoor test of the water collection device. a) Schematic of the water collection device. b) Optical images of the device at different time points. c) Real‐time variations in solar flux, outdoor temperature, and humidity during the test. d) Ion concentrations of seawater and collected water.

Regarding practical implementations, the water collection performance is also an important criterion for evaluation that has been largely ignored in many previous studies on salt rejection evaporators.^[^ ^29^ ^]^ In this study, we prepared a portable solar water purification prototype to demonstrate its potential for practical seawater desalination (**Figure** **6**a). A total of 11 MG evaporators, each containing eight macrochannels, were inserted into insulation foam and floated on seawater. The prototype was deployed on a rooftop in Shan Shui Po, and the evaluation period spanned from 8:00 to 18:00. Under natural sunlight irradiation conditions, vapor was generated and condensed at the transparent cover to form droplets, which were eventually collected at the bottom of the device (Figure 6b). The average sunlight flux was ≈0.68 kW m^−2^, and the highest recorded outdoor temperature was 33 °C (May 29, 2023; Figure 6c). After a 1‐d test, 25.69 g of water is collected. Based on the evaporator area of 34.54 cm^2^, the daily water collection rate is 7.44 L m^−2^. This freshwater productivity is superior to the previously recorded salt‐rejection solar evaporators ((≈2.5 L m^−2^)^[^ ^60^ ^]^ and (≈5 L m^−2^)^[^ ^29^ ^]^). Furthermore, over 99.9% of the ions are removed by the solar evaporation process, and the ion concentration of the purified water is lower than the requirements of the World Health Organization (WHO) and Environmental Protection Agency (EPA) (Figure 6d). This excellent water production and purification performance per square meter of our evaporator satisfies the daily drinking water requirements of three individuals (2 L and 2.5 L per d for females and males, respectively, as recommended by the European Food Safety Authority).^[^ ^61^ ^]^

## 3 Conclusion

In summary, we demonstrate a highly efficient and salt rejecting solar evaporator in both high‐salinity NaCl solution and seawater. The excellent performance is attributed to the joint contribution of the MG architecture and the macrochannels: the former enables localized salt crystallization in the low‐temperature zone and exhibits enhanced light absorption (≈99.6%) and evaporative surface (>30%), and the latter connects the high‐salt zone with bulk water to enhance the natural convection flow for salt rejection while minimizing heat loss caused by convection flow. In addition, a daily water production of ≈7.4 L m^−2^ under outdoor conditions is achieved, highlighting the practical potential for providing fresh water for off‐grid regions.

## Conflict of Interest

The authors declare no conflict of interest.

## Supporting information

Supporting Information

Supplemental Video 1

## Data Availability

The data that support the findings of this study are available in the supplementary material of this article.
