# Supplementary material for: Simultaneous Salt Rejection and Heat Localization Via Engineering Macrochannels in Morning Glory‐Shaped 3D Evaporator
Source: Adv Sci (Weinh). 2024 Aug 29;11(40):2405639. doi: 10.1002/advs.202405639 (PMC11515903; doi:10.1002/advs.202405639)
Supplement: Supplementary file 1 — Supporting Information [file ADVS-11-2405639-s002.docx]

Supplementary Materials for

**Simultaneous salt rejection and heat localization via engineering macrochannels in morning glory-shaped 3D evaporator**

Zhengyi Mao, Yicheng Han, Junda Shen, Lei Zhang, Youneng Xie, Jiahua Liu, Haikun Wu, Zhen Yu Xiaoguang Duan, Yaoxin Zhang, Jian Lu

**This PDF file includes:**

Supplementary Methods

Supplementary Figure. 1 to 23

References

# S1. Supplementary Methods

## Materials

Polyurethane (PU) emulsion and crosslinker-2500 were purchased from ShunDe SanSheng Trade Co., Ltd, China. Carbon black was purchased from Carbot Co. Ltd.

## Preparation of MG evaporator

PU emulsion and crosslinker were first mixed with deionized (DI) water at a volumetric ratio of 1 : 0.05 : 1.5. Then, 1 wt% carbon black was added to the mixture, and the as-prepared solutions were poured into customized silica moulds and frozen at −80 °C for 24 h and then freeze-dried at −50 °C for 48 h to obtain highly interconnected evaporator.

## Mechanical test

The compression tests were conducted using a mechanical testing device (Cellscale, Canada) in water. A cuboid evaporator with a length of 20 mm and a height of 8 mm was prepared. The strain rate was set as 1 mm s^−1^, and the compression/recovery process was conducted 100 times.

## Evaporation performance

A solar simulator (Newport Class AAA Solar Simulator 94043A) equipped with an air mass 1.5G optical filter was used as the light source. The mass changes during evaporation were recorded on an electronic balance (Zhuojing, China). All evaporation tests were measured at a temperature of 24 ± 1°C and a humidity of 60%. The concentrated seawater was prepared by evaporation of natural seawater under 80℃.

## Characterization

The reflectance (R) was obtained using an ultraviolet-visible light–near-infrared spectrometer (Solidspec-3700). The absorptance of the evaporator (α) was obtained by the direct-hemispherical reflectance (α = 1 −R). Infrared images were captured using an infrared camera (Fluke Ti400, USA). The microstructures of the evaporators and the salt crystals were characterized using a scanning electron microscope (Quanta 450 FEG, FE) operated at 10 kV. The samples were coated with a layer of gold to increase their conductivity (Q150 T S, Quorum Technologies Ltd.) The wettability of the samples was measured using a Krüss DSA100 contact-angle goniometer at room temperature and under ~60% relative humidity. For the DSC measurement, the samples were placed in an Al crucible and measured with a linear heating rate of 5 °C /min, under nitrogen flow flux (50 mL/min), within the temperature range from 25 to 150 °C. All optical images and videos were recorded using a digital camera (Eos 5D Mark IV, Canon, Japan). The salinity was measured using a digital refractometer (HI 96801, Hanna Instruments).

## Multiphysics thermo-fluid flow model for salt rejection

The salt transport of dilute species, laminar fluid flow and heat transfer of evaporators were studied through COMSOL 6.5. The continuity, momentum, energy and advection equations were coupled and numerically solved by the finite element method (FEM). Here, different porosities were employed to distinguish the wick domain ($\varepsilon=0.7$) and marcochannels ($\varepsilon=1$) according to experimental characterization. Both wick and marcochannels were filled with brine of 3.5 wt % salt concentration as the initial condition. The buoyancy-driven flow was captured by introducing the gravity effect due to concentration and temperature differences. Here, the density of brine spatially varies with concentration $c$, given as $\rho\left( c \right)=\rho_{0}+\beta c$, where $\rho_{0}$ denotes the density of water and $\beta$ is a constant. Differently, the temperature effect was implemented by applying the Boussinesq approximation in the buoyancy term, given as $\rho\mathbf{g}\left[ 1-\alpha(T-T_{\infty}) \right]$ in the momentum conservation, where $\alpha$ is the thermal expansion coefficient.

A uniform evaporation flux was employed on the upper surface of the evaporator. Meanwhile, a fresh brine supply was applied to the bottom boundary to balance the evaporation loss. For the salt transport process, a mass flux of salt species was applied on the upper boundary, simulating the salt accumulation with respect to the corresponding water evaporation. All the sidewalls were considered no-slip and no-flux boundaries.

Figures S6 (a) and (b) depict the velocity fields of the MG evaporator with marcochannels under different top surface concentrations. It was found that capillary penetration dominates with 3.5 wt% salt concentration at the top surface. In comparison, flow direction changes in the marcochannels as concentration increases, suggesting a significant buoyancy-driven flow occurs and capillary penetration is suppressed. It should be noted that the length scale of marcochannels is much larger than that of hydraulic pores, resulting in that gravity being sufficiently strong to dominate the fluidic behavior instead of capillary force. In addition, this buoyancy-driven flow triggers the convective salt reflow from the surface to marcochannels, significantly accelerating the salt rejection. The Peclet number quantitively depicted the ratio of contribution between convection and diffusion (see Figure S7), given as $Pe=\frac{L\mathbf{u}}{D}$, where *L*, **u** and *D* denote the characteristic length, flow velocity and diffusivity, respectively.

# Supplementary Figures and Tables


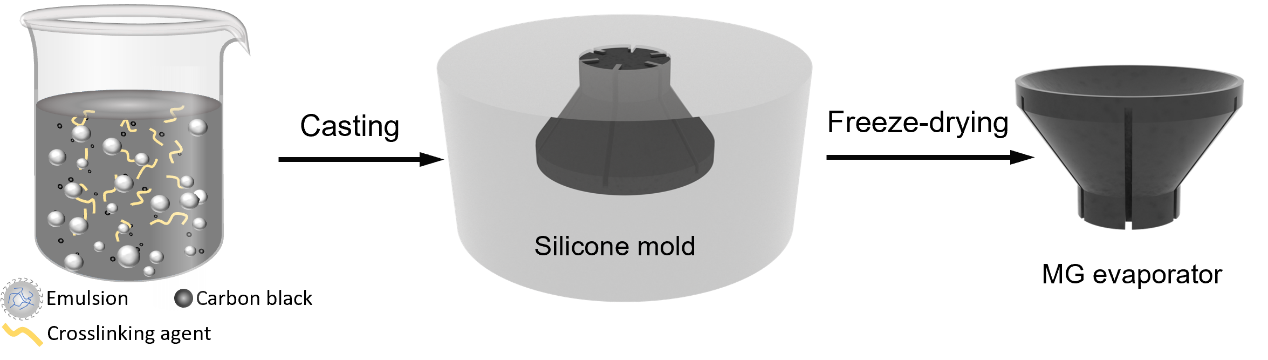


Figure S1. Preparation process of our evaporator.


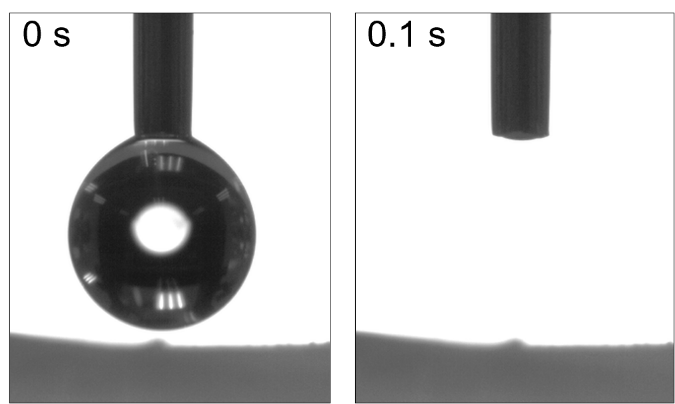


Figure S2. Contact angel of the prepared PU/carbon black evaporator. The contact angle is about 0°. The volume of the droplet is 800 µL.


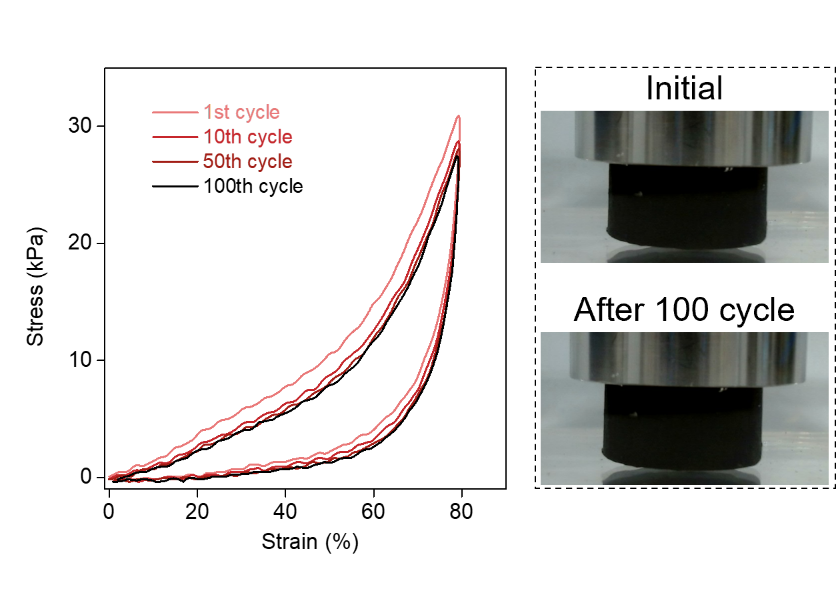


Figure S3. Mechanical performance of the evaporator. The right is the optical images of materials before and after 100 cyclic compressions.


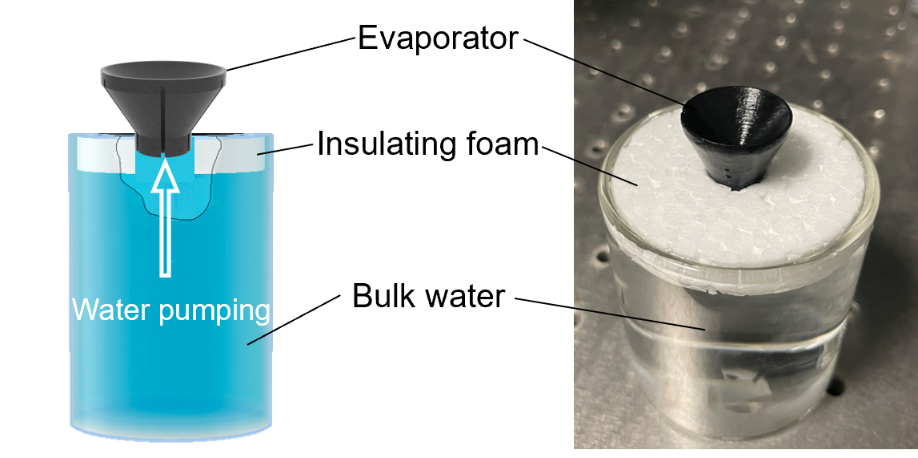


Figure S4. The evaporator was inserted into the EPS foam. Water can be pumped from the hole in the foam through capillary force. The foam was cut with a custom cutter to have the same diameter as the container, to minimize evaporation from the gap between the foam and the container.


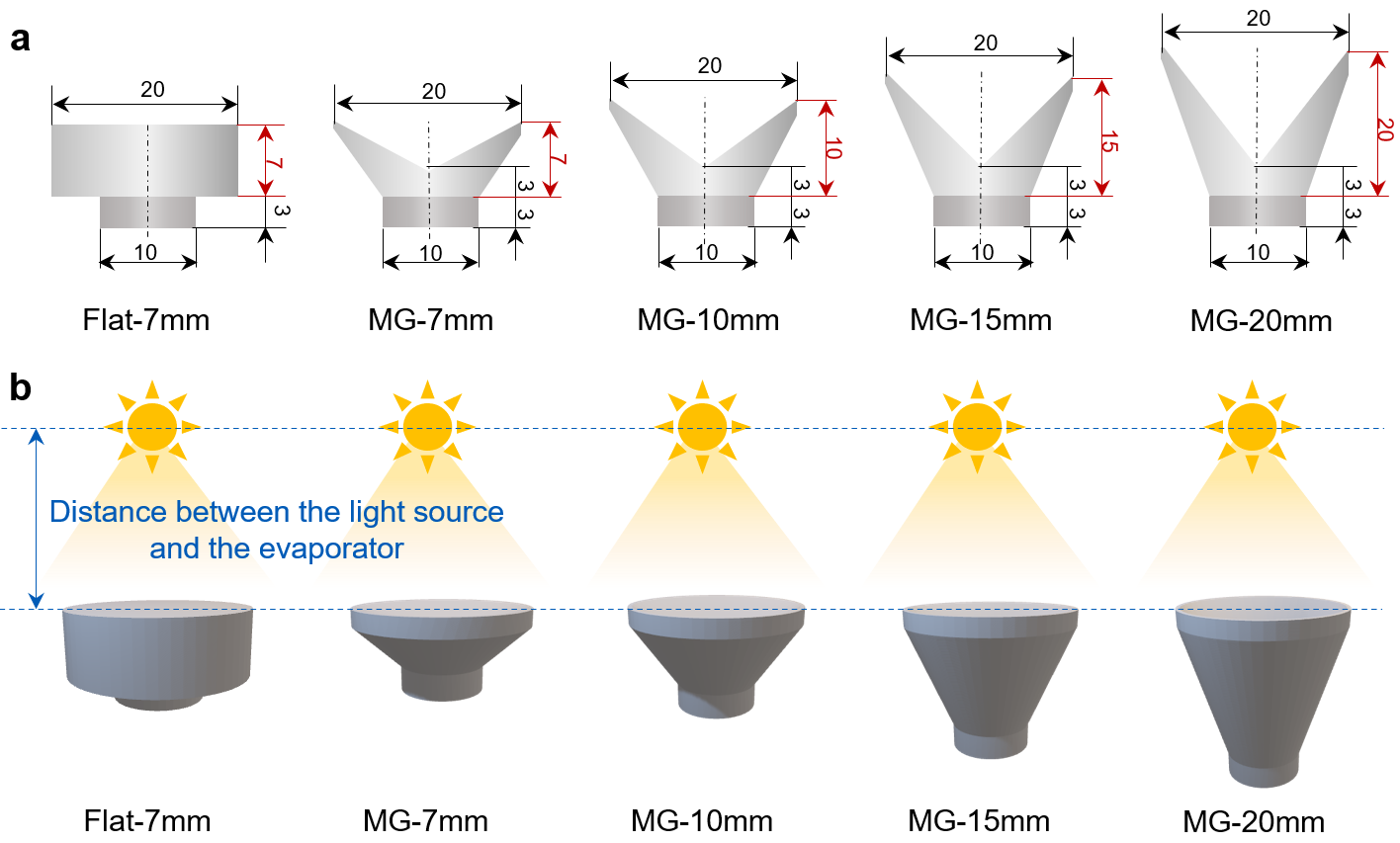


Figure S5. (a) Structure design of the evaporators with different heights. Unit: mm. To ensure consistent testing conditions, the distance between the top surface of each evaporator and the light source was maintained at the same level (b).


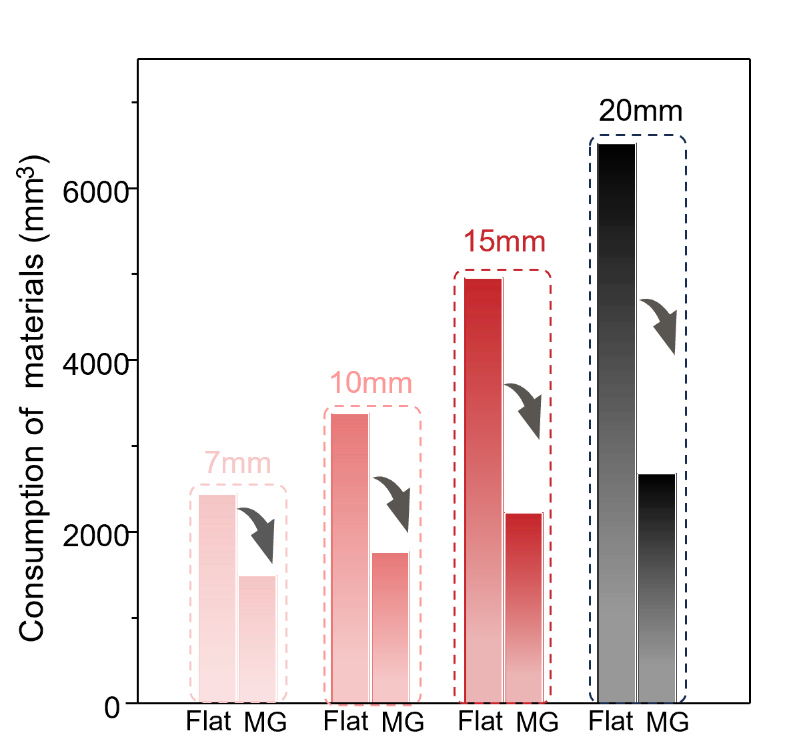


Figure S6. Comparison of materials consumed for flat and MG evaporators with the same height.


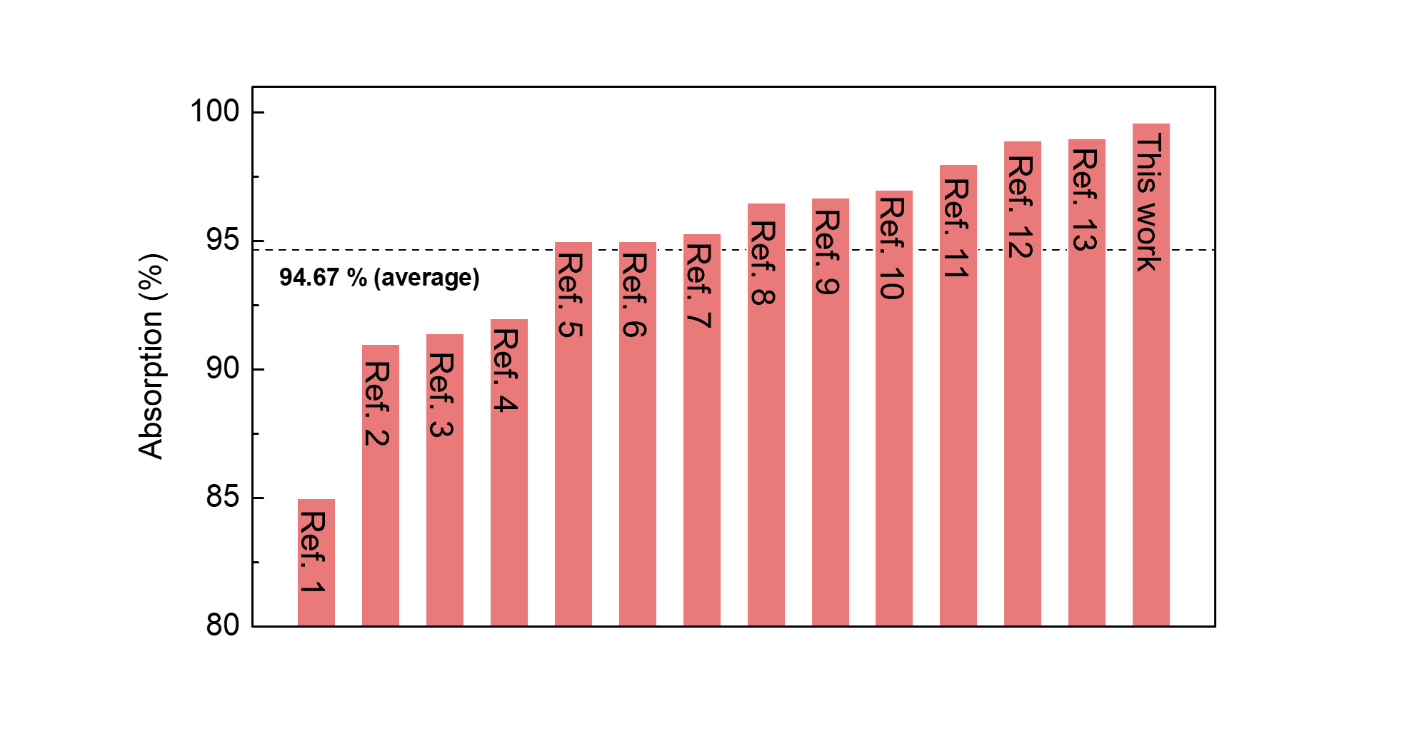


Figure S7. Comparison on the light absorption of our MG evaporator with previously reported works.^[1–13]^


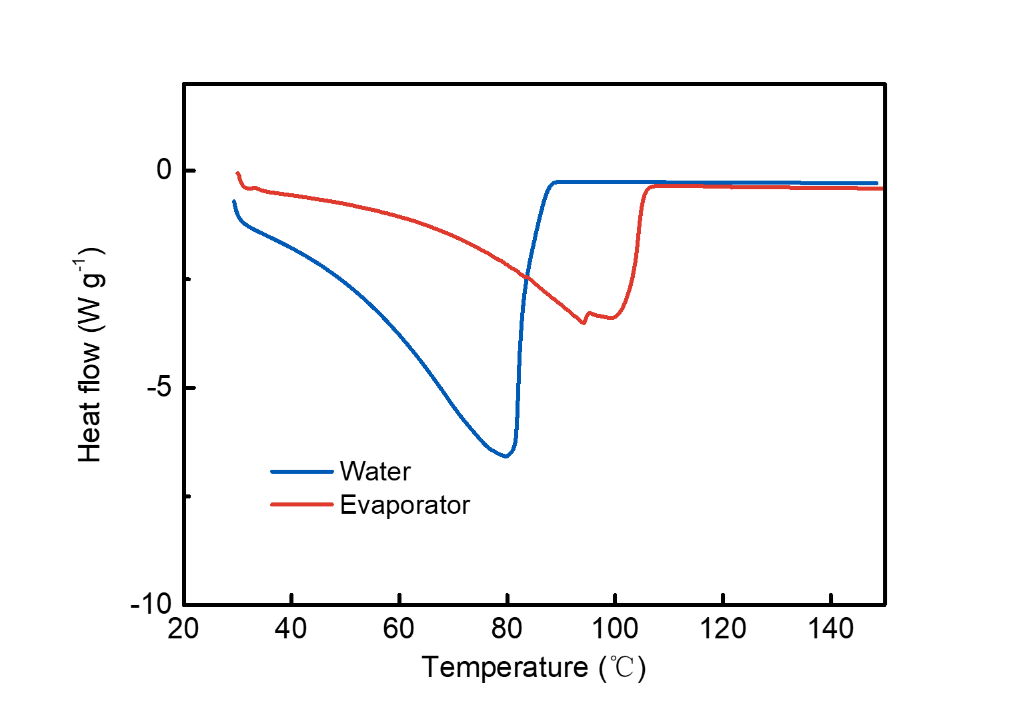


Figure S8. DSC curves of pure water and the evaporator. The measured evaporation enthalpy of pure water and water in the evaporator is 2475 J/g and 1517 J/g, respectively. The water evaporation enthalpy of the evaporator is much smaller than that of pure water, due to the influence of the polymer network on the evaporation process.^[14,15]^

*
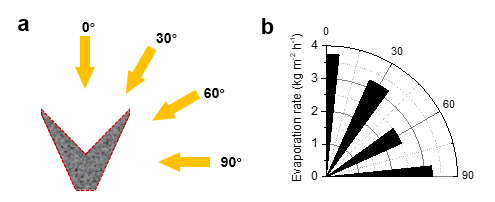
*

Figure S9. Evaporation performance of MG-20 with different illumination angles. (a) Schematic illustration of the illumination angle. (b) Evaporation rate of the MG-20 with different illumination angles.


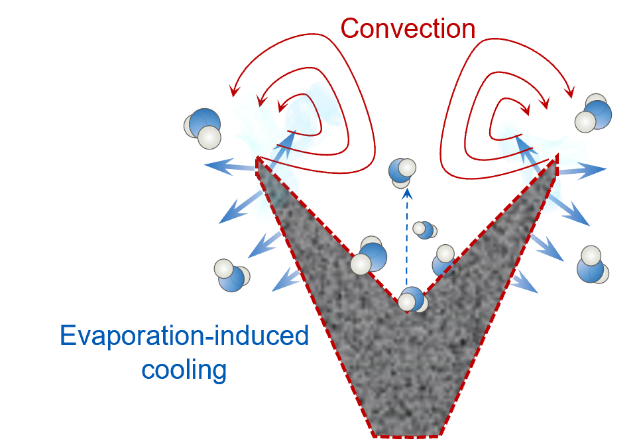


Figure S10. Schematic illustration of gradient temperature distribution of the inner surface.


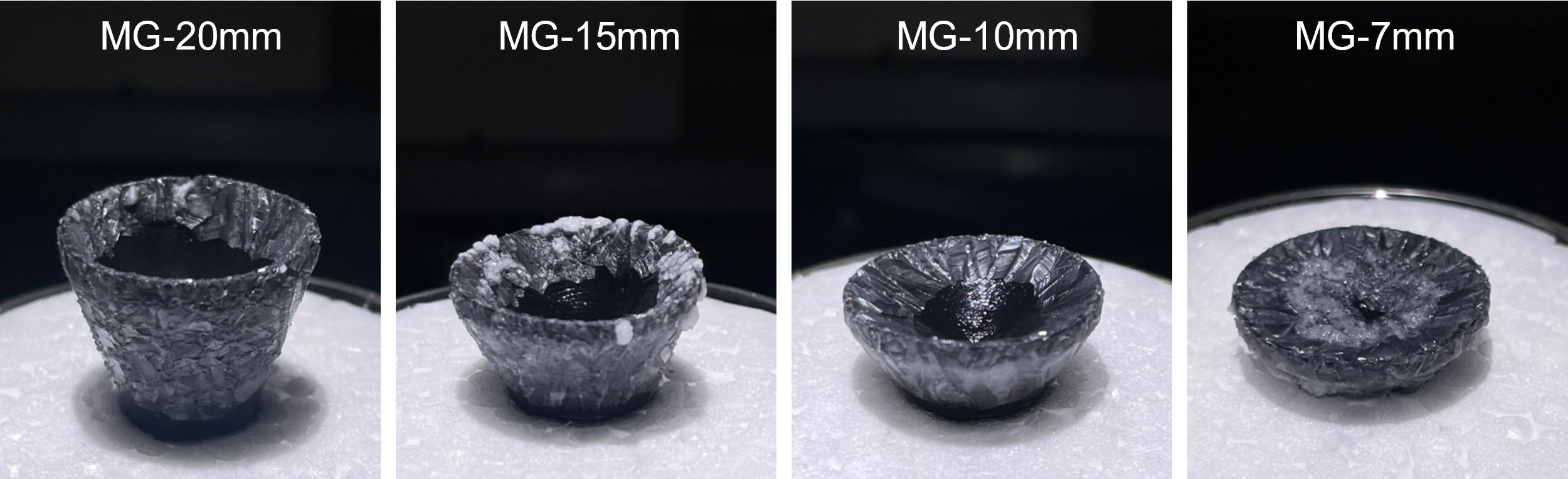


Figure S11. Salt crystallization on the MG evaporators.


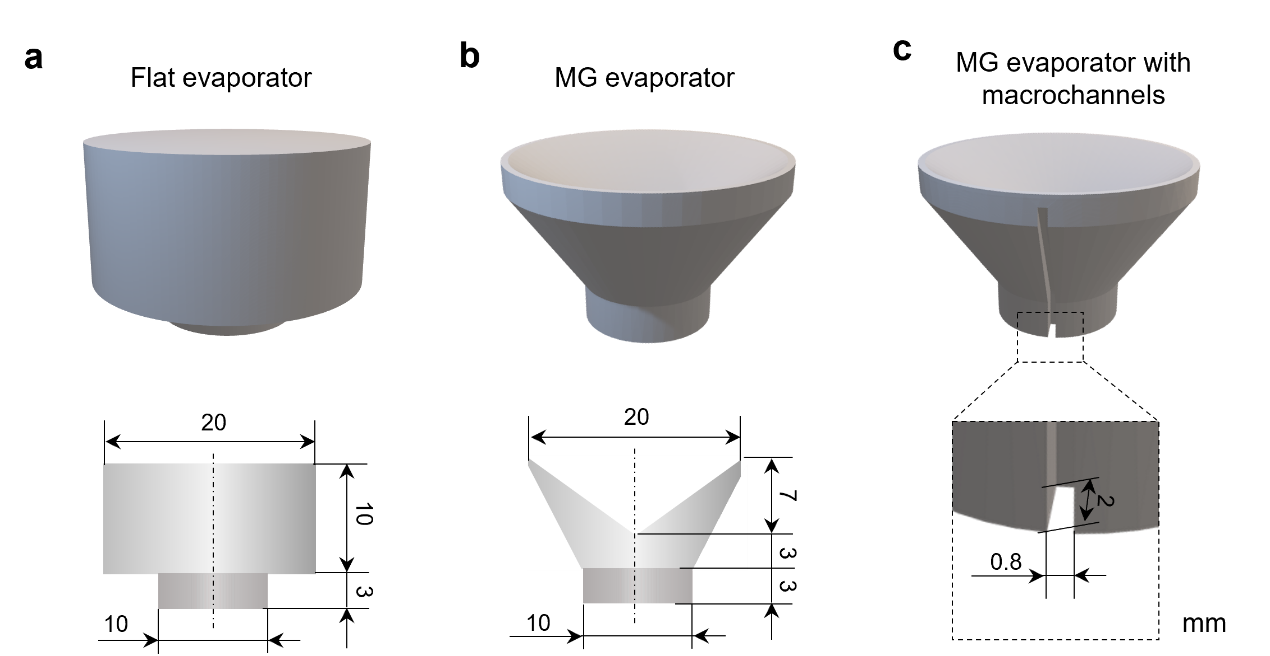


Figure S12. The detailed structure of the (a) flat evaporator, (b) MG evaporator and (c) MG evaporator with macrochannels (unit: mm). The size of 0.8 mm for the macrochannel was selected based on a consideration of the capillary force, convective flow, and manufacturing feasibility. When the diameter is too large, capillary forces are insufficient to provide adequate water supply. According to the formula L_max_=4γcosθ/ρgh, the maximum size of the macrochannel ranges from 1.22 mm for self-water pumping to 20 mm. At the same time, a higher evaporator requires a smaller macrochannel. However, excessively large size of the macrochannel increases convection, leading to increased heat loss through convective flow. Considering manufacturing complexity, structures with excessively small diameters significantly escalate production costs. Therefore, we opted for a 0.8 mm diameter as a proof of concept.


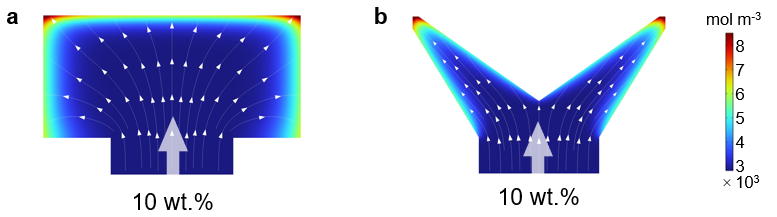


Figure S13. Simulated salt transport pathways of the flat and MG evaporators. The edge-preferential capillary flow and intense water evaporation facilitate the accumulation of salt ions at the edge, while the low temperature at the edge promotes salt nucleation. Consequently, this process results in the edge-preferential salt crystallization.

*
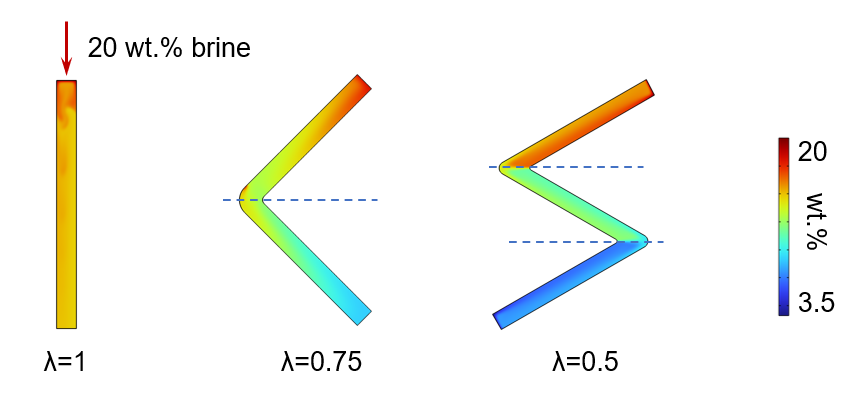
*

Figure S14. Salt transfer in macrochannels with different tortuosity.

To investigate the impact of tortuosity on salt rejection, simulations were performed using macrochannels with different tortuosity of 1, 0.75 and 0.5. In simulations, the concentrated and warm saline (20 wt.%, 35$℃$) and the bulk seawater (3.5 wt.%, 25$℃$) were added to the upper and lower boundaries, respectively. The concentration profiles after 1800s are shown in Figure S14. The low tortuosity macrochannel rapidly transports salt ions from the top surface to the bottom, whereas the increased tortuosity slows down salt transfer efficiency, resulting in a lower salt concentration on the bottom surface. The macrochannel with a tortuosity of 0.5 displays a triphasic concentration distribution due to the hindrance of mass transfer by the tortuous structure.


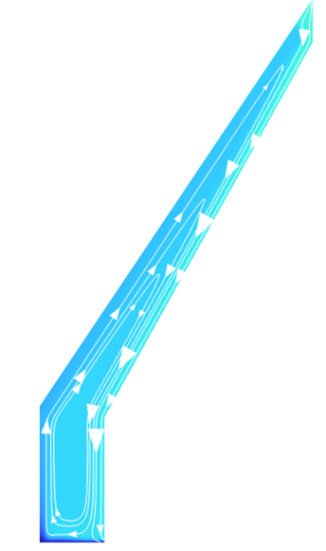


Figure S15. Magnified the convective ionic circulation path in the macrochannel.


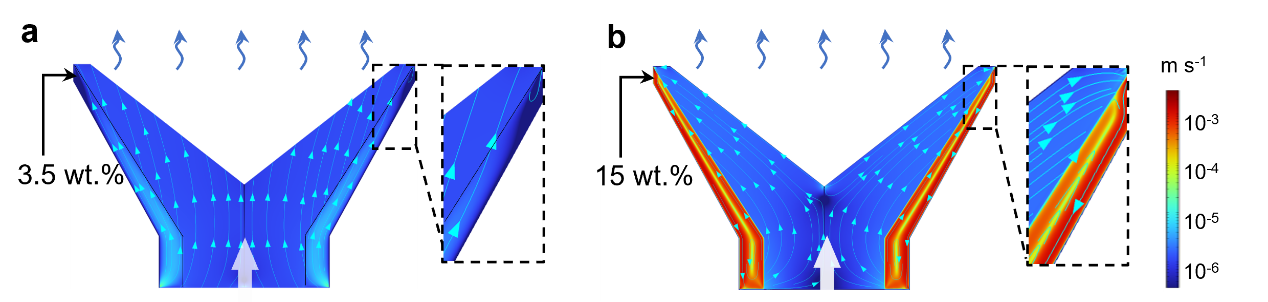


Figure S16. Buoyancy-driven natural convection in macrochannels. Simulated velocity fields for the salt flow inside the wick structure and macrochannels for the two different top concentrations: (a) 3.5 wt% and (b) 15 wt%. Buoyancy-driven natural convection was triggered by the salt gradient in the macrochannel in (b), which fast reflow the salt ions to bulk water.


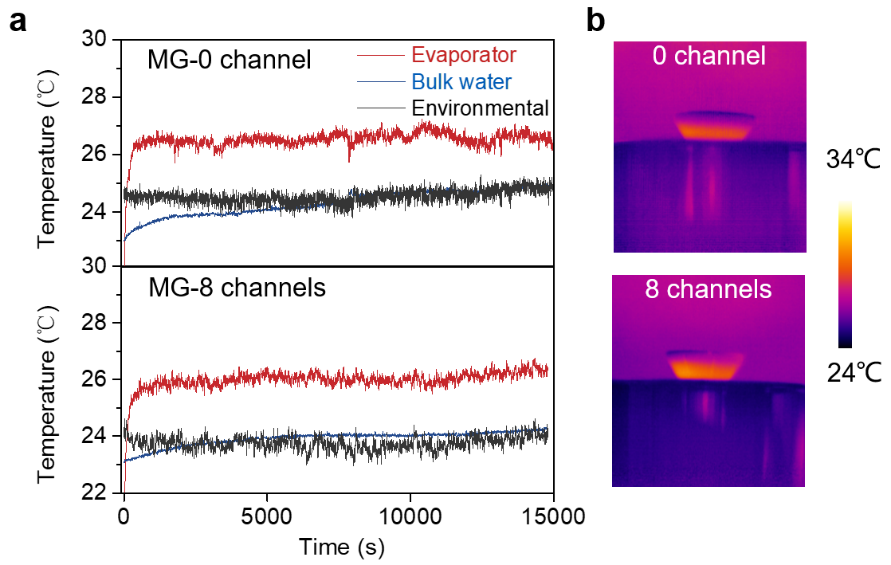


Figure S17. (a) Temperature variations at different positions of the evaporation system during 4 h of evaporation. (b) IR images of the MG evaporator and MG evaporator with 8 channels after 4 h of illumination under 1 sun.


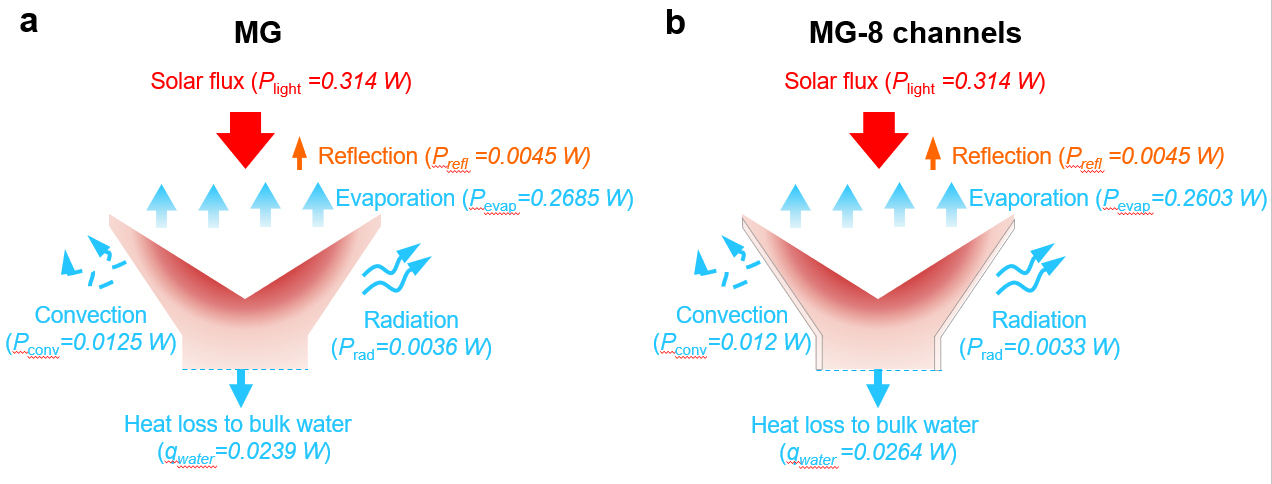


Figure S18. Calculated heat flow in MG (a) and MG-8 channels (b).

Since the temperature of the evaporator is higher than the temperature of the surroundings, there is no energy input from the environment. Thus, the energy consumed by evaporation (*P*_evap_) can be calculated as ^[16]^,

*P*_evap_ = *P*_light_ − *P*_environment_

Here, the light input power *P*_light_ = *αC*_opt_*q*_i_, where α is the optical absorption coefficient; *C*_opt_ is the optical concentration; and *q*_i_ is the normal direct solar irradiation (1 kW m^−2^ for 1 sun at AM 1.5). The energy loss to the environment *P*_environment_ can be calculated as:

*P*_environment_ = *P*_conv_ + *P*_rad_ + *P*_cond_ = *h(T_2_ − T_1_) + εσ(T*_2_^4^*−T*_1_^4^*)* + *q*_water_

where *T*_2_ is the temperature of the evaporation surface, *T*_1_ is the environment temperature, *h* is the convection heat transfer coefficient, *ε* denotes the optical emission, *σ* is the Stefan–Boltzmann constant, and *q*_water_ denotes the energy transferred to the bulk water. Given that the convective and radiative heat transfers to the bulk water are negligible, for simplicity, the energy loss to the environment by convection can be described as *P*_conv_ = *h*(*T*_2_ − *T*_1_), the radiative loss to the surroundings is *P*_rad_ = *εσ(T*_2_^4^*−T*_1_^4^*)*. After calculation, the energy flux of the MG evaporator and MG evaporator with 8 macrochannels was plotted in Fig. R1. The heat loss to the bulk water of the MG is calculated to be 0.0239W, while the heat loss of the MG-8 is 0.0264W. The convection of the 8 macrochannels in the MG-8 slightly increases the heat loss to the bulk water by 0.0025W, which accounts for 0.8% of the total solar energy input.

*
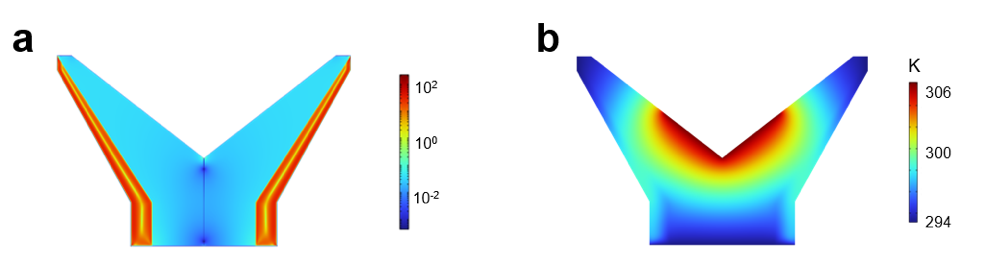
*

Figure S19. Simulated Peclet number for heat transfer (a) and temperature distribution (b) of the MG evaporator with macrochannel.

To discuss the heat loss from the convective flow in the, we conducted heat transport simulations in the macrochannel and wick structure. The results show that there is indeed heat convection in the macrochannel, which inevitably carries away some heat (Figure S19a). However, due to our design of separating the high-salt and high-temperature zones, only a slight heat loss is caused by the convection. No significant temperature difference was observed between the macrochannel and wick structure, implying that the convection has not led to a substantial heat loss (Figure S19b).


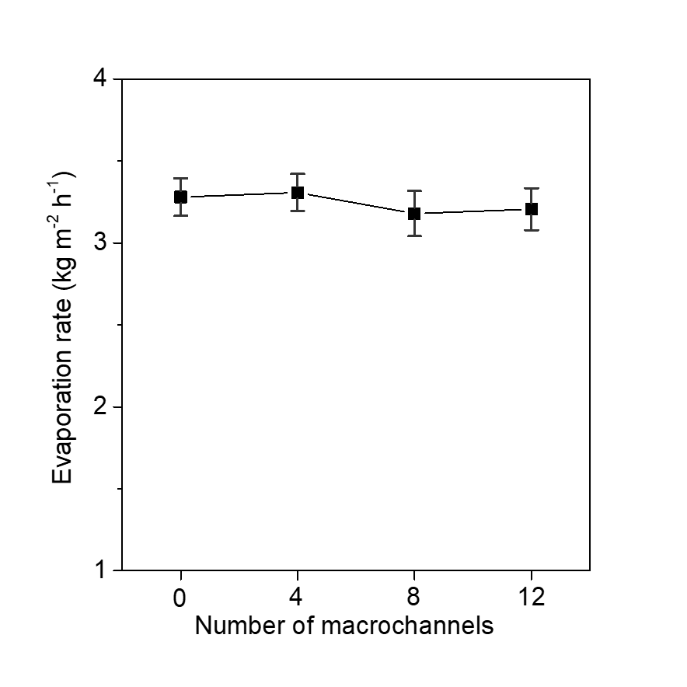


Figure S20. The evaporation rate of the MG evaporators with the different number of macrochannels.


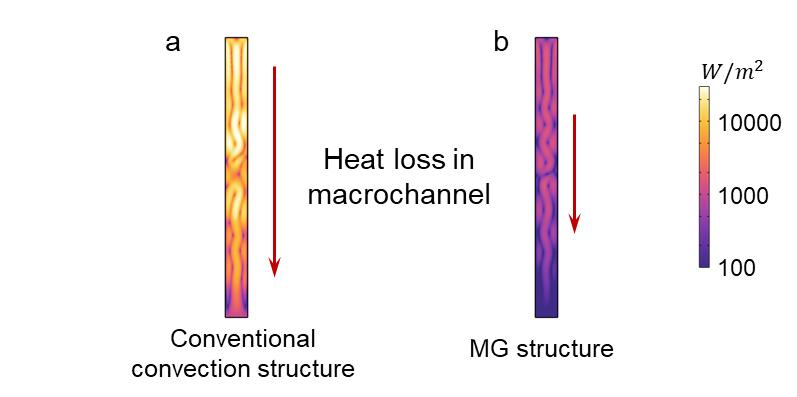


Figure S21. Simulated heat loss in the macrochannel of (a) conventional convection structure and (b) MG structure.

To demonstrate the effect of isolating the high-salt zone and the high-temperature zone on reducing heat loss, we conducted simulations for comparison. Two simulations with non-isolated high-temperature zone and isolated high-temperature zone in the same marcochannel were developed. In both simulations, concentrated saline (20 wt.%) and the bulk seawater (3.5 wt.%, 25$℃$) were added to the upper and lower boundaries, respectively. The same evaporation rate was applied to the upper boundaries. To imply the separation of high-temperature zone, we simulated the conventional convection salt-resistant structure by applying a Dirichlet boundary condition of 35.5$℃$ to the upper boundary, where the high-temperature zone and high-salt zone were not isolated (Figure 21a). Instead, a Dirichlet boundary condition of 26.5 $℃$ was applied for our strategy, indicating an environment where the high-temperature zone was isolated (Figure S21b). The total heat flux exhibits a drastic reduction with isolating the high-temperature zone, indicating that the heat loss is significantly suppressed.


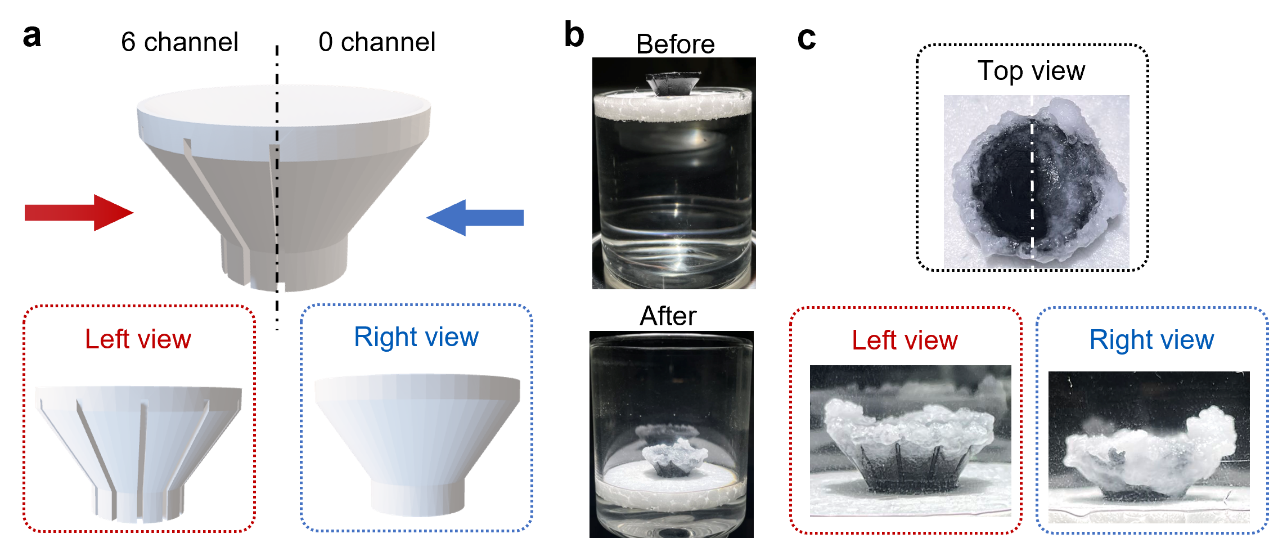


Figure S22. (a) Illustration of the MG evaporator with one section containing 6 macrochannels and another section without any macrochannels. (b) The height of the seawater before and after 150 h continuous evaporation. (c) Optical images of the evaporator after operation.


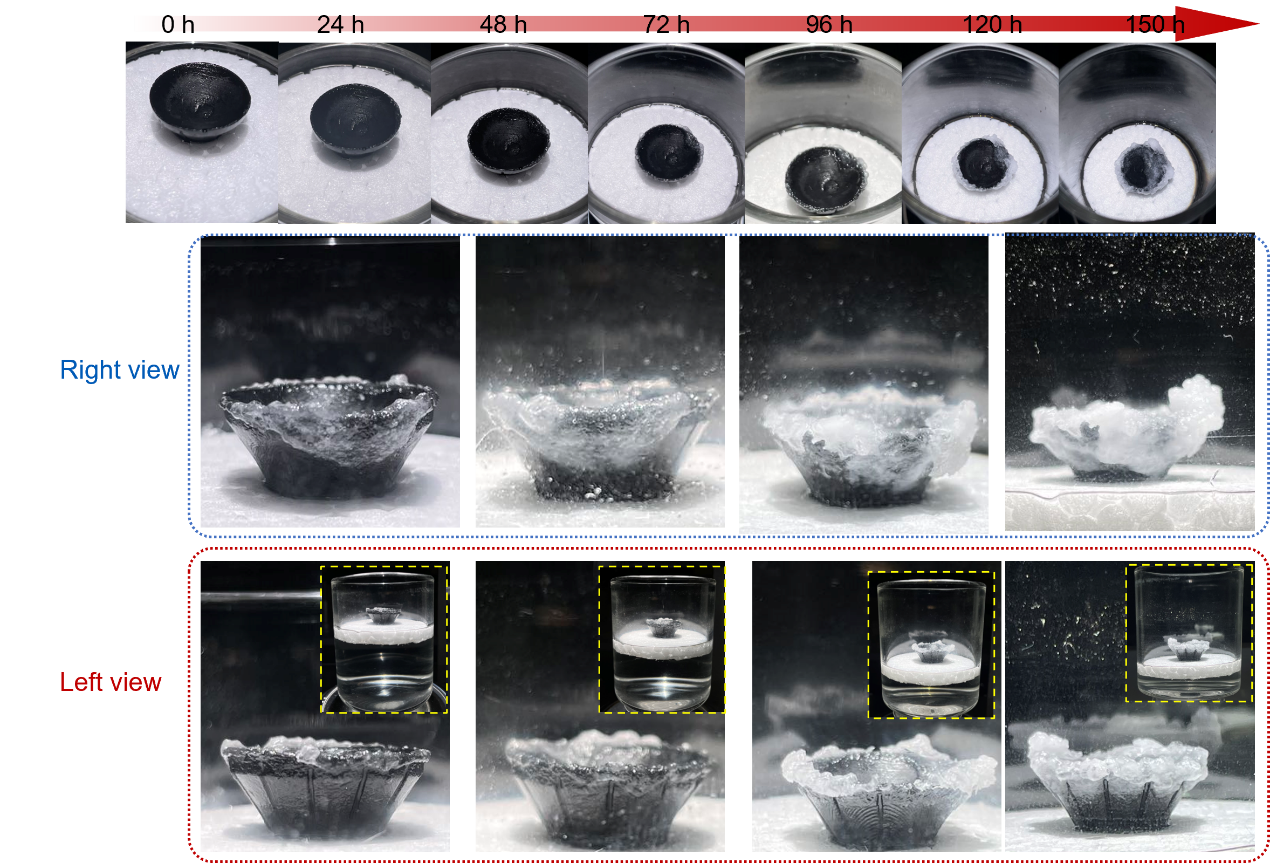


Figure S23. Detailed optical images of the evaporator and the system during 150 h continuous operation. 110 mL seawater was nearly fully evaporated after the continuous illumination. The section without macrochannels (right view) was fully covered by a dense salt crust, which prevents light absorption and vapor generation. However, the section with macrochannels (left view) shows a localized salt crystallization. The fast convective flow in the macrochannel enables the salt fast backflow to the bulk water, avoiding salt precipitating on the main surface.

**References**

[1] C. Ma, J. Yan, Y. Huang, C. Wang, G. Yang, *Sci. Adv.* **2018**, *4*, DOI 10.1126/sciadv.aas9894.

[2] H. Zhang, L. Li, B. Jiang, Q. Zhang, J. Ma, D. Tang, Y. Song, *ACS Appl. Mater. Interfaces* **2020**, *12*, 16503.

[3] X. Dong, L. Cao, Y. Si, B. Ding, H. Deng, *Adv. Mater.* **2020**, *32*, 1908269.

[4] H. Zhang, L. Li, N. He, H. Wang, B. Wang, T. Dong, B. Jiang, D. Tang, *EcoMat* **2022**, *4*, e12216.

[5] Y. Wang, C. Wang, X. Song, M. Huang, S. K. Megarajan, S. F. Shaukat, H. Jiang, *J. Mater. Chem. A* **2018**, *6*, 9874.

[6] Y. Zhang, Y. Wang, B. Yu, K. Yin, Z. Zhang, *Adv. Mater.* **2022**, *34*, 2200108.

[7] L. Zhu, L. Sun, H. Zhang, H. Aslan, Y. Sun, Y. Huang, F. Rosei, M. Yu, *Energy Environ. Sci.* **2021**, *14*, 2451.

[8] S. Chaule, J. Hwang, S. Ha, J. Kang, J. Yoon, J. Jang, *Adv. Mater.* **2021**, *33*, 2102649.

[9] C. Wang, Y. Wang, W. Guan, P. Wang, J. Feng, N. Song, H. Dong, L. Yu, L. Sui, Z. Gan, L. Dong, *J. Colloid Interface Sci.* **2022**, *612*, 88.

[10] Y. Xu, Z. Guo, J. Wang, Z. Chen, J. Yin, Z. Zhang, J. Huang, J. Qian, X. Wang, *ACS Appl. Mater. Interfaces* **2021**, *13*, 27129.

[11] Y. Sui, D. Hao, Y. Guo, Z. Cai, B. Xu, *J. Mater. Sci.* **2020**, *55*, 298.

[12] Z. Sun, C. Han, S. Gao, Z. Li, M. Jing, H. Yu, Z. Wang, *Nat. Commun.* **2022**, *13*, 5077.

[13] W. Xu, X. Hu, S. Zhuang, Y. Wang, X. Li, L. Zhou, S. Zhu, J. Zhu, *Adv. Energy Mater.* **2018**, *8*, 1702884.

[14] X. Zhou, F. Zhao, Y. Guo, B. Rosenberger, G. Yu, *Sci. Adv.* **2019**, *5*, eaaw5484.

[15] Y. Guo, X. Zhou, F. Zhao, J. Bae, B. Rosenberger, G. Yu, *ACS Nano* **2019**, *13*, 7913.

[16] H. Song, Y. Liu, Z. Liu, M. H. Singer, C. Li, A. R. Cheney, D. Ji, L. Zhou, N. Zhang, X. Zeng, Z. Bei, Z. Yu, S. Jiang, Q. Gan, *Adv. Sci.* **2018**, *5*, 1800222.
